# Radiologic drivers of dysphagia after intracerebral haemorrhage: a flexible endoscopic evaluation of swallowing-guided retrospective analysis on intensive care unit

**DOI:** 10.1186/s42466-026-00498-0

**Published:** 2026-05-12

**Authors:** Samra Hamzic, Omar Alhaj Omar, Linda Danielczik, Stefan T. Gerner, Maxime Viard, Michael Bender, Heidrun H. Kraemer, Priyanka Boettger, Martin Juenemann, Tobias Braun

**Affiliations:** 1 Department of Neurology, Justus-Liebig-University, Klinikstrasse 33, 35392 Giessen, Germany; 2 Translational Neuroscience Network Giessen (TNNG), Giessen, Germany; 3 Justus-Liebig-University Giessen, Giessen, Germany; 4 Swiss University of Speech and Language Sciences-hlo, St. Gallen, Switzerland; 5 Center of Mind, Brain & Behavior (CMBB), Marburg, Germany; 6 Department of Neurology, University Hospital, Erlangen, Germany; 7 Department of Neurology, Kantonsspital Winterthur, Winterthur, Switzerland; 8 Department of Neurosurgery, Justus-Liebig-University, Giessen, Germany; 9 Department of Cardiology, Justus-Liebig-University, Giessen, Germany; 10 Department of Neurology, Lahn-Dill-Kliniken Wetzlar, Wetzlar, Germany

**Keywords:** Dysphagia, Intracerebral haemorrhage, ICU, FEES, Swallowing disorders

## Abstract

**Introduction:**

Dysphagia is a frequent and clinically relevant complication after intracerebral haemorrhage (ICH) and is associated with increased morbidity and mortality. While radiologic severity and lesion characteristics are known to influence neurological outcome, their relationship with objectively measured dysphagia severity (DS) and swallowing management remains insufficiently defined. This study investigated the association between radiologic ICH burden and DS assessed by flexible endoscopic evaluation of swallowing (FEES), and examined the clinical impact of FEES-guided swallowing management in an neuro intensive care unit (ICU) cohort.

**Methods:**

We conducted a retrospective analysis of consecutive ICH patients treated in neurological ICU who underwent standardized bedside dysphagia assessment, with FEES performed when clinically indicated and feasible. DS and functional oral intake were quantified using the Fiberoptic Endoscopic Dysphagia Severity Scale (FEDSS) and the Functional Oral Intake Scale (FOIS-G). Multivariable regression models adjusted for age and clinical severity were used to examine associations between radiologic parameters (hematoma volume, location, ICH score, intraventricular and subarachnoid haemorrhage) and dysphagia outcomes.

**Results:**

Of 241 patients 120 (49.8%) were diagnosed with relevant dysphagia FEES was performed in 68 patients (28.2%) at a median of 13 days after admission. After FEES, the diet was modified in 67% of patients and the oral intake score improved from a median of one to four. Radiologic severity was independently associated with dysphagia outcomes: higher ICH score predicted lower odds of of favourable FOIS-G and FEDSS scores. Deep intracerebral haemorrhage, intraventricular haemorrhage and subarachnoid haemorrhage were associated with worse functional oral intake. FEES led to a modification of swallowing management in 67% of examined patients, most frequently allowing safer and more liberal oral intake compared with bedside assessment alone.

**Conclusion:**

In ICU-treated patients with ICH, dysphagia is common and closely linked to radiologic severity and lesion characteristics. FEES frequently reveals clinically relevant discrepancies between bedside assessment and actual swallowing function and substantially influences swallowing management. Integrating radiologic risk stratification with FEES-guided decision-making may improve safety, nutrition, and rehabilitation planning in this high-risk population.

## Introduction

While dysphagia following ischemic stroke has been extensively investigated, dysphagia after intracerebral haemorrhage (ICH) remains comparatively underexplored despite similarly high prevalence rates, reported to affect up to 60% of patients in the acute stage [1, 2]. Available evidence suggests that dysphagia after ICH is associated with larger hematoma volumes, deep or infratentorial hemorrhage location, intraventricular extension, and higher clinical severity [3, 4, 5]. hese factors are thought to impair swallowing through mass effect, altered consciousness, hydrocephalus, and disruption of cortical–subcortical swallowing networks. Lesions involving the insular cortex, internal capsule, basal ganglia, and thalamus contribute to swallowing deficits, as well [3, 6, 7]. Neuroimaging studies emphasize the role of fronto-insular circuits in deglutition, with lobar haemorrhages in these regions linked to impaired motor control of swallowing [3, 8]. Nevertheless, most studies in ICH-related dysphagia have relied on bedside screening tools, clinical judgement, or surrogate outcomes such as feeding-tube placement, which may inadequately capture true swallowing physiology. *Recent studies have highlighted the prognostic relevance of simple clinical and CT-based markers in spontaneous intracerebral haemorrhage, particularly hematoma volume and intraventricular extension; however, the swallowing consequences of these injury patterns remain insufficiently defined *[12,13]*.*

Oropharyngeal dysphagia can be evaluated either via clinical screening procedures or via Flexible Endoscopic Evaluation of Swallowing (FEES) and/or Videofluoroscopic Swallow Study (VFSS).

Bedside screening tools are not capable of reliably detecting silent aspiration [14]. Although VFSS is considered an instrumental gold standard for comprehensive swallowing assessment, its application is often limited in acute care settings due to logistical and medical constraints [15].

FEES has emerged as a gold-standard diagnostic tool in neurogenic dysphagia, particularly in critically ill patients [1, 16, 17, 18]. It enables direct visualization of dysphagic symptoms, and airway protection at the bedside and can be safely repeated. Importantly, FEES is not merely diagnostic but directly informs clinical decision-making regarding oral intake, diet texture, and therapeutic strategies [1, 16].

In neurological intensive care unit (ICU) settings, clinical dysphagia assessment alone may either overestimate or underestimate swallowing impairment, leading to unnecessarily restrictive nil-per-os orders or, conversely, unsafe oral intake [1,16]. FEES frequently reveals clinically relevant discrepancies between bedside assessment and objective swallowing function, with direct consequences for nutrition, airway safety, and rehabilitation planning [1,16].

Only few studies have systematically applied instrumental swallowing diagnostics to ICH cohorts, and even fewer have examined dysphagia severity (DS) in direct relation to radiologic injury patterns [3, 4, 5].

Against this background, the present study follows a unified conceptual framework linking radiologic ICH severity, FEES-based dysphagia characterization, and FEES-guided swallowing management. The primary aim was to investigate the association between radiologic characteristics of ICH (including hematoma volume, location, intraventricular and subarachnoid extension, and composite severity scores) and DS assessed by FEES. A secondary aim was to evaluate how FEES modifies swallowing management beyond bedside assessment alone, as reflected by changes in functional oral intake.

By integrating routinely available neuroimaging parameters with instrumental swallowing diagnostics, this study seeks to provide clinically applicable risk stratification for dysphagia after ICH. Such an approach may support early and targeted dysphagia management not only in specialized neuro-ICUs, but also in acute care environments where neurological expertise is limited [19].

## Methods

We retrospectively analysed patients’ routine data between the period of April 2014 to February 2021 that were treated for ICH on the neurological and neurosurgical intensive care unit in the university hospital Giessen, Germany.

### Patients

All ICH patients treated in our intensive care unit that received dysphagia assessment (DA) were included in the analysis. Patients without at least DA were not included in the analysis. For acquisition and use of data for scientific analyses, an ethical approval was obtained from the local ethical committee of the Justus Liebig University in Giessen, Germany (identifier: AZ 220/21 and AZ 208/16).

We assessed patients’ demographics, modified Rankin scale (mRS) prior to bleeding event, admission status (National Institutes of Health Stroke Scale (NIHSS), Glasgow Coma Scale (GCS), risk factors, antithrombotic treatment, oral anticoagulation), type of intracerebral haemorrhage (deep haemorrhage, lobar haemorrhage, intraventricular haemorrhage, presence of subdural haematoma or subarachnoid haemorrhage or haemorrhagic transformation following ischemic stroke), anatomic characteristics (haematoma volume as measured by ABC/2 method [20], side, affected lobes), ICH-score [21], Graeb-score in the event of intraventricular haemorrhage [22], medical interventions (reversal of oral anticoagulation, surgical treatment of the haemorrhage, placement of external ventricular drainage). Haematoma expansion was defined as haematoma volume increase of ≥ 6 ml or 33% between two brain scans. As outcome parameters, we documented medical status at discharge (NIHSS, mRS, Barthel index, mortality, necessity and duration of mechanical ventilation). We also assessed dysphagia associated outcome parameters such as diagnosis of dysphagia, FEES procedure, placement of a nasogastric or parenteral feeding tube, tracheotomy, pneumonia, presence of nasogastric or parenteral feeding tube at discharge, severity of dysphagia as measured by the Fiberoptic Endoscopic Dysphagia Severity Score (FEDSS) [17] and route of feeding before and after fees examination as measured by Functional Oral Intake Scale, German version (FOIS-G) [23]. We also documented length of hospital stay and time from admission to FEES examination.

### Dysphagia examination

All patients received a bedside clinical evidence-based DA performed by experienced speech and language therapists [24]. DA evaluated vigilance, voice quality, voluntary cough, saliva handling. Trial swallows were conducted where clinically appropriate. Based on DA results, patients were categorized regarding swallowing safety. Appropriate diet as well as a a Functional Oral Intake Scale score (FOIS-G, German version) were assigend [23]. FOIS-G as a seven-tier score assesses the oral intake of liquids and food. Lower scores on this scale indicate worse dysphagia symptoms.

FEES was indicated when DA suggested relevant dysphagia, when clinical findings were inconclusive, or when neurological severity, radiologic burden, or prolonged intubation raised concern for impaired swallowing safety. When feasible, FEES was conducted to objectively evaluate swallowing function. FEES feasibility was defined by hemodynamic stability, sufficient vigilance, absence of prohibitive nasal or pharyngeal pathology, and the ability to tolerate endoscopic examination. Consequently, FEES was more frequently performed in patients with suspected dysphagia who were clinically stable enough to undergo instrumental assessment, introducing an unavoidable selection toward less severely unstable patients.

FEES has become the gold standard of endoscopic evaluation of swallowing, especially in neurogenic cohorts. We performed FEES following a standardised examination protocol of our ICU [25]: A small endoscope (3,6 mm in diameter), was introduced through the inferior nasal meatus and the epipharynx in the mesopharynx. The swallowing of saliva and different consistencies of food and liquids, penetration, aspiration, localization and amount of residues as well as patients’ reactions (such as coughing) were visualised and documented. By definition, penetration is entering of any material into the airway (above the level of the vocal folds), aspiration means entering of any material below the level of the vocal folds. In the first step of the procedure, anatomical changes, handling of saliva and the movement of swallowing related structures were tested. Then, we tested liquid, puree, and solid food. All consistencies were applied three times. If a consistence appeared unsafe to test, we skipped the consistence. Using the findings in FEES, the appropriate oral diet was chosen for the patient. All FEES procedures were performed or supervised by an experienced investigator. Severity of dysphagia was assessed with the FEDSS score. FEDSS is a 6-tiered dysphagia severity score; higher scores define more severe dysphagia symptoms [17].

Dysphagia was defined as a FOIS-G < 7 score or a FEDSS ≥ 2 score after FEES.

### Statistical methods

All statistical analyses were conducted using the SPSS software package version 29 (www.spss.com). Descriptive statistics were employed to summarize data on demographic, clinical and radiologic characteristics. To assess the normality of the distribution of continuous variables, a Shapiro–Wilk test was conducted. Normally distributed data are presented as mean (SD) and compared using two sided t-tests, and non-normally distributed data are presented as median (range) and compared using the Mann–Whitney U-test and Wilcoxon signed-rank test, respectively. The level of significance was set at alpha = 0.05. To investigate patient characteristics associated with dysphagia, we used multivariate regression analysis, adjusting for *mRS before admission* and age, with FEDSS and FOIS-G treated as median-split binary variables. This regression analysis was used to evaluate the impact of various parameters on the worsening of FEDSS and FOIS-G scores. *Forest plots for were created in R 4.4.0 using RStudio to visualize the regression analysis results.*

## Results

The baseline characteristics of the 241 patients are summarized in Table 1 The mean age was 72.5 ± 13.5 years, and 44% were women. At admission, the median NIHSS (0–42) was 12 (IQR 5–23) and the median Glasgow Coma Scale was 13 (IQR 9–15). Premorbid disability was low (median pre-mRS 0, IQR 0–1). Vascular history and medications were common: prior stroke in 24.1%, atrial fibrillation in 31.5%, and documented hypertension in 85.5%; 17.8% were taking direct oral anticoagulants, 7.1% vitamin K antagonists, and 21.6% antiplatelet monotherapy.

**Table 1:** Baseline characteristics and outcome parameters of the overall cohort, stratified into normal swallowing status

Baseline characteristics	Cohort (n = 241)	Normal swallowing function (n = 121)	Relevant Dysphagia (n = 120)	*P*-Value
Age,^a^ years	72.5 (13.5)	73.5 (13.8)	71.4 (13.2)	.231
Sex,^b^ female	106 (44)	55 (45.5)	51 (42.0)	.646
**Admission status**				
NIHSS at admission (0—42)^c^	12 (5—23)	10 (3—24)	13 (8—20)	.045^*^
Glasgow coma scale at admission^c^	13 (9—15)	14 (7—15)	13 (10—15)	.832
Pre-mRS^c^	0 (0—1)	0 (0—0)	0 (0—1)	.167
Previous stroke^b^	58 (24.1)	22 (18.2)	36 (30)	.032^*^
Atrial fibrillation^b^	76 (31.5)	42 (34.7)	34 (28.3)	.289
Blood pressure^b^	206 (85.5)	103 (85.1)	103 (85.8)	.877
Direct oral anticoagulants^b^	43 (17.8)	31 (25.6)	12 (10)	.001
Vitamin K Antagonists^b^	17 (7.1)	6 (5.0)	11 (9.2)	.204
Antithrombotic monotherapy^b^	52 (21.6)	22 (18.2)	30 (25)	.200
**Type of the intracranial haemorrhage**				
Deep intracerebral haemorrhage	107 (44.4)	52 (43.0)	55 (45.8)	.657
Lobar intracerebral haemorrhage^b^	108 (44.8)	58 (47.9)	50 (41.7)	.330
Intraventricular haemorrhage^b^	115 (47.7)	61 (50.4)	54 (45.0)	.402
Subdural hematoma^b^	13 (5.4)	7 (5.8)	6 (5.0)	.802
Subarachnoid haemorrhage^b^	50 (20.7)	21 (17.4)	29 (24.2)	.194
Haemorrhagic transformation after ischemic stroke^b^	38 (15.8)	20 (16.5)	18 (15)	.746
**Characteristic of the haemorrhage**				
Left sided^b^	143 (59.3)	70 (57.9)	74 (61.7)	.547
Right sided^b^	112 (46.5)	60 (49.6)	52 (43.3)	.332
Both sides^b^	23 (9.5)	11 (9.1)	12 (10)	.811
Lobar haemorrhage in frontal lobe^b^	47/108 (43.5)	26 (21.5)	21 (17.5)	.437
Lobar haemorrhage in parietal lobe^b^	47/108 (43.5)	24 (19.8)	23 (19.2)	.896
Lobar haemorrhage in temporal lobe^b^	39/108 (36.1)	20 (16.5)	19 (15.8)	.884
Lobar haemorrhage in occipital lobe^b^	39/108 (36.1)	20 (16.5)	19 (15.8)	.884
Haemorrhage in posterior fossa^b^	36 (14.9)	21 (17.6)	15 (12.5)	.292
Graeb-Score^c^	0 (0—3)	1 (0—3)	0 (0—2)	.149
ICH-Score^c^	1 (1—3)	2 (1—3)	1 (1—2)	.247
Hematoma volume (cm^3^)^a^	28.6 (38.1)	24.2 (29.2)	32.8 (44.9)	.084
Rebleeding^b^	29 (12)	15 (12.4)	14 (11.7)	.863
Hematoma Expansion^a^	5.2 (19.6)	4.6 (17.0)	5.9 (21.9)	.597
**Interventions**				
EVD^b^	12 (5)	2 (1.7)	10 (8.3)	.018^*^
Surgical operation^b^	53 (22)	11 (9.1)	42 (35.0)	<.001^*^
Reversal of the anticoagulation^b^	31 (12.9)	17 (14.1)	14 (11.7)	.582
**Outcome parameter**				
NIHSS at discharge^c^	5 (2—11)	2 (0—4)	10 (5—14)	<.001^*^
mRS at discharge^c^	5 (3—6)	4 (2—5)	5 (4—6)	.740
Barthel index at discharge^c^	25 (10—60)	60 (30—85)	10 (5—30)	<.001^*^
In-hospital mortality^b^	77 (32)	23 (19.2)	54 (44.6)	<.001^*^
Ventilation necessary^b^	102 (42.3)	47 (38.8)	55 (45.8)	.274
Duration of ventilation, hours^c^	180 (48—456)	46.2 (0—48)	192.1 (0—300)	<.001^*^

^a^mean ± SD.

^b^n (%).

^c^median (interquartile range: 25th-75th percentile).

EVD, external ventricular drain; interquartile range; mRS, modified Rankin scale (0 no deficit to 6 death); NIHSS, National Institutes of Health Stroke Scale (ranging from 0, no deficit, −40, severe neurological deficit; 40 is maximum because in comatose ataxia is not scored); SAH, subarachnoid haemorrhage.

Data are presented as mean ± SD, n (%), or median (interquartile range, 25th–75th percentile), as appropriate. Relevant dysphagia was defined as FOIS-G < 7 on dysphagia assessment and/or FEDSS ≥ 2 when FEES was performed. P values refer to between-group comparisons of the normal swallowing function and relevant dysphagia subgroups. Although formally statistically significant p values are reported where applicable, due to the retrospective and exploratory design of the study these results should be interpreted with caution and should not be regarded as confirmatory evidence

Hemorrhage types were evenly split between deep intracerebral hemorrhage (44.4%) and lobar intracerebral hemorrhage (44.8%); intraventricular hemorrhage occurred in 47.7%, subdural hematoma in 5.4%, subarachnoid hemorrhage in 20.7%, and hemorrhagic transformation after ischemic stroke in 15.8%. Laterality was left-sided in 59.3%, right-sided in 46.5%, and bilateral in 9.5%. Among lobar events (n = 108), distributions were frontal 43.5%, parietal 43.5%, temporal 36.1%, and occipital 36.1%. Posterior fossa hemorrhage was present in 14.9%. The median Graeb score was 0 (IQR 0–3) and the median ICH score 1 (IQR 1–3). Mean hematoma volume was 28.6 ± 38.1 cm^3^, with rebleeding in 12% and mean hematoma expansion of 5.2 ± 19.6 cm^3^.

Interventions included external ventricular drain placement in 5%, surgical hematoma evacuation or related procedures in 22%, and anticoagulation reversal in 12.9%. Outcomes at discharge showed a median NIHSS of 5 (IQR 2–11), median mRS of 5 (IQR 3–6), and median Barthel Index of 25 (IQR 10–60). In-hospital mortality was 32%. Mechanical ventilation was required in 42.3% for a median of 180 h (IQR 48–456).

Dysphagia-related scales showed low baseline oral intake that improved following instrumental assessment. The FOIS-G score before FEES was a median of 1 (interquartile range [IQR] 1–2); after FEES the median was 4 (IQR 1–5). After DA, the FOIS-G median was 3 (IQR 1–7). The median FEES-based dysphagia severity score (FEDSS) was 3 (IQR 2–6). Based on the results of the FEES, 46 patients (67%) had an adjustment of their diet to match their swallowing abilities, resulting in a change of the FOIS-G-Score. This resulted in 5 Patients that needed more restrictions in their diet and 41 patients that had their initial restrictions lowered Table 2.

**Table 2:** Dysphagia-related parameters (n = 241)

Dysphagia parameters:	Cohort (n = 241)
Dysphagia^b^	120 (49.8)
FEES conducted^b^	68 (28.2)
Time (days) untill FEES^c^	13 (6—22.5)
Outcome parameter	
PEG^b^	40 (16.6)
NGT^b^	112 (46.5)
Tracheostomy^b^	28 (11.6)
Pneumonia^b^	105 (43.6)
Discharge with PEG or NGT^b^	54 (22.4)
FOIS-G before FEES^c^	1 (1—2)
FOIS-G after FEES^c^	4 (1—5)
FOIS-G DA^c^	3 (1—7)
FEDSS^c^	3 (2—6)

^a^mean ± SD

^b^n (%)

^c^median (interquartile range: 25th-75th percentile)

### Hemorrhage characteristics and adjusted associations with dysphagia

To examine factors associated with dysphagia, we ran multivariable logistic regression models adjusted for age and premorbid mRS. Analyses were restricted to patients with a clinical diagnosis of dysphagia. FEDSS and FOIS-G were dichotomized at their median values and coded so that the dependent variable reflected good status (i.e., higher FOIS-G; lower FEDSS); adjusted odds ratios (aORs) therefore represent the odds of a good outcome.

**Fig. 1:**
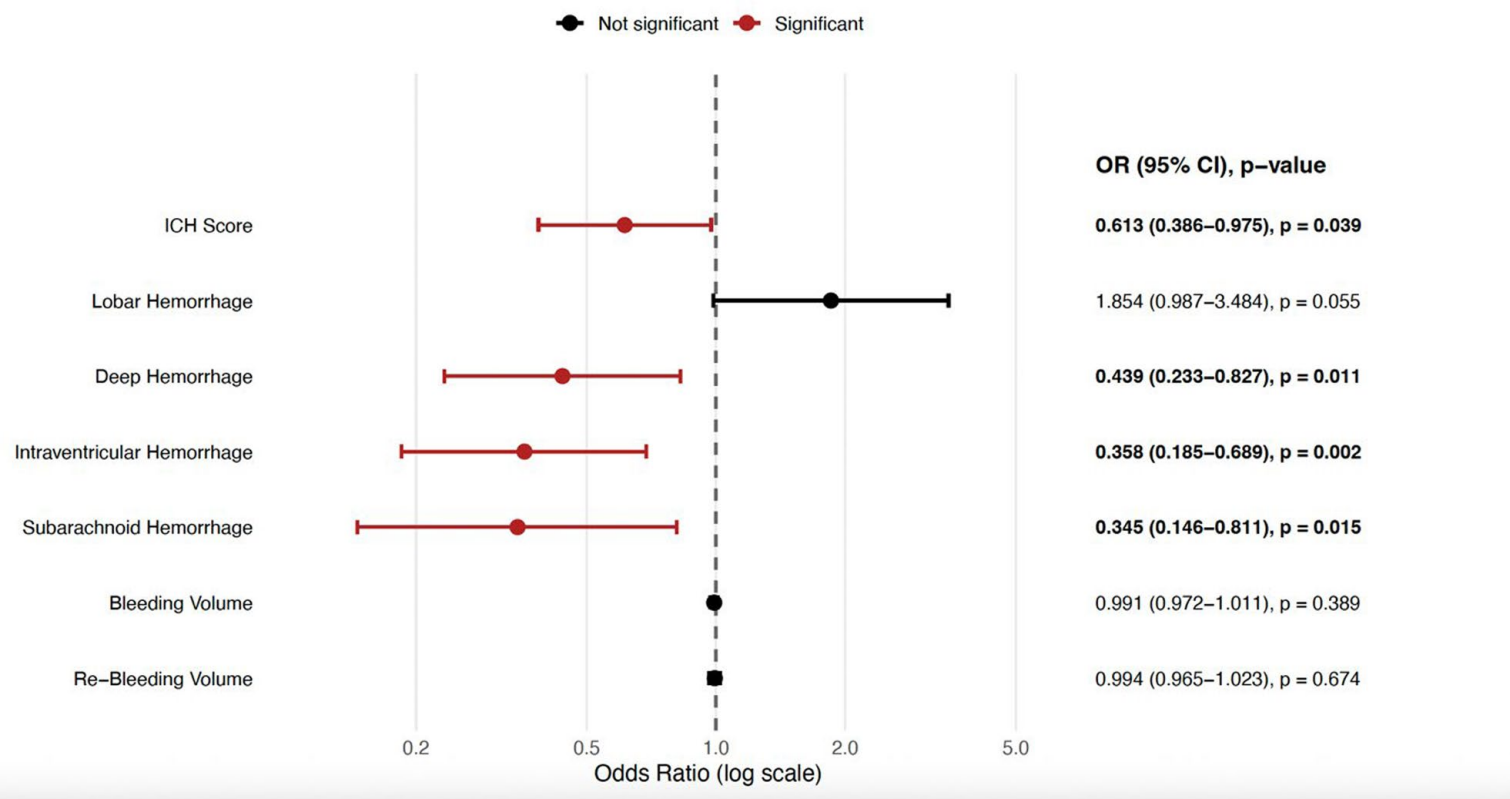
Forest plot of predictors of good outcome (FOIS-G). Points show odds ratio and horizontal lines show 95% confidence intervals

In the dysphagia cohort, for FOIS-G (good outcome) after the dysphagia assessment, a higher ICH score was independently associated with lower odds of a good outcome (aOR 0.613, 95% CI 0.386–0.975; p = 0.039). Deep haemorrhage location, subarachnoid haemorrhage (SAH), and intraventricular haemorrhage (IVH) were each linked to lower odds of a good FOIS-G after assessment (deep: aOR 0.439, 95% CI 0.233–0.827; p = 0.011; SAH: aOR 0.345, 95% CI 0.146–0.811; p = 0.015; IVH: aOR 0.358, 95% CI 0.185–0.689; p = 0.002) (*refer to *Fig. 1)*.*

**Fig. 2:**
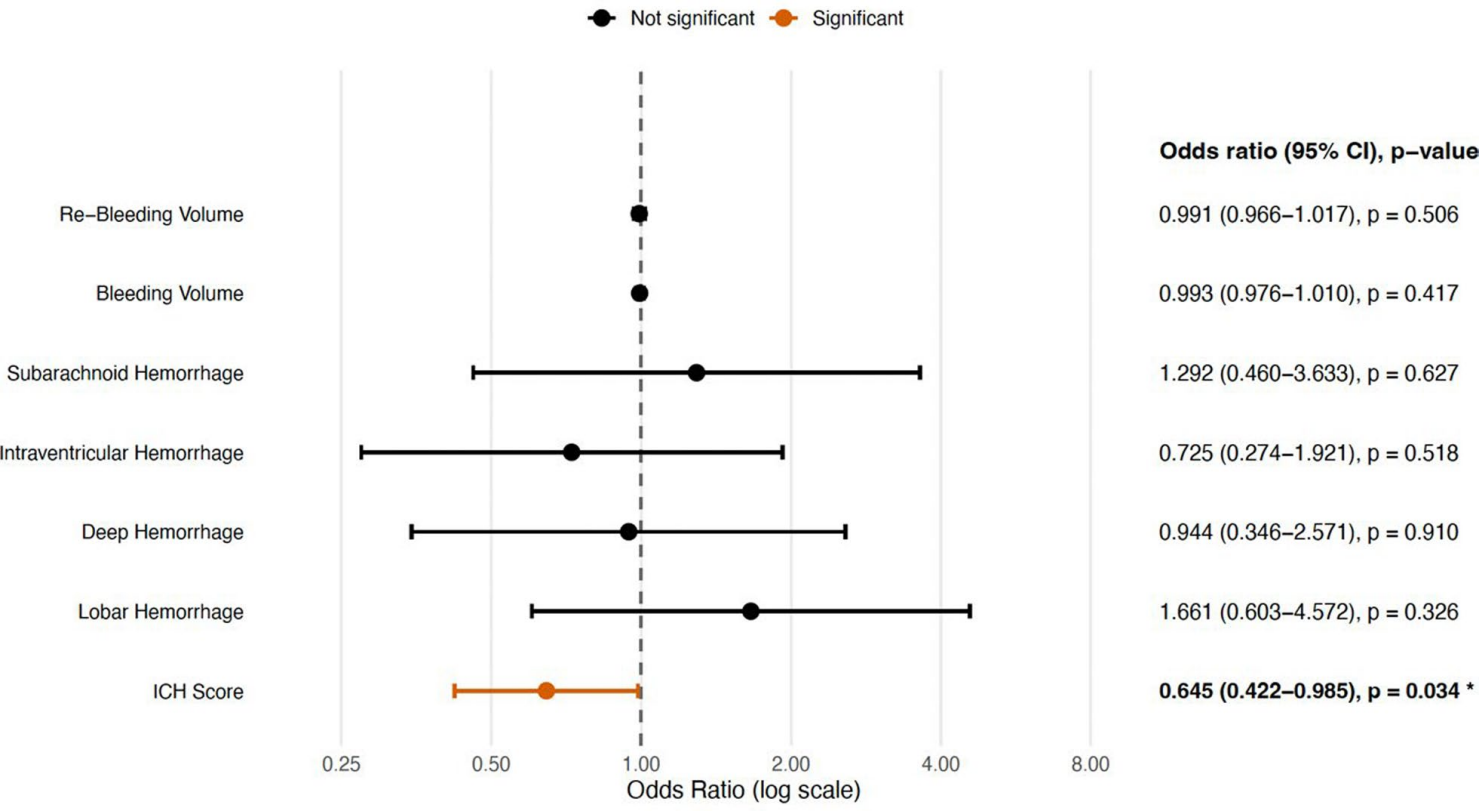
Forest plot of predictors of good outcome (FOIS-G after FEES). Significant results (p < 0.05) are highlighted in orange and marked with *

**Fig. 3:**
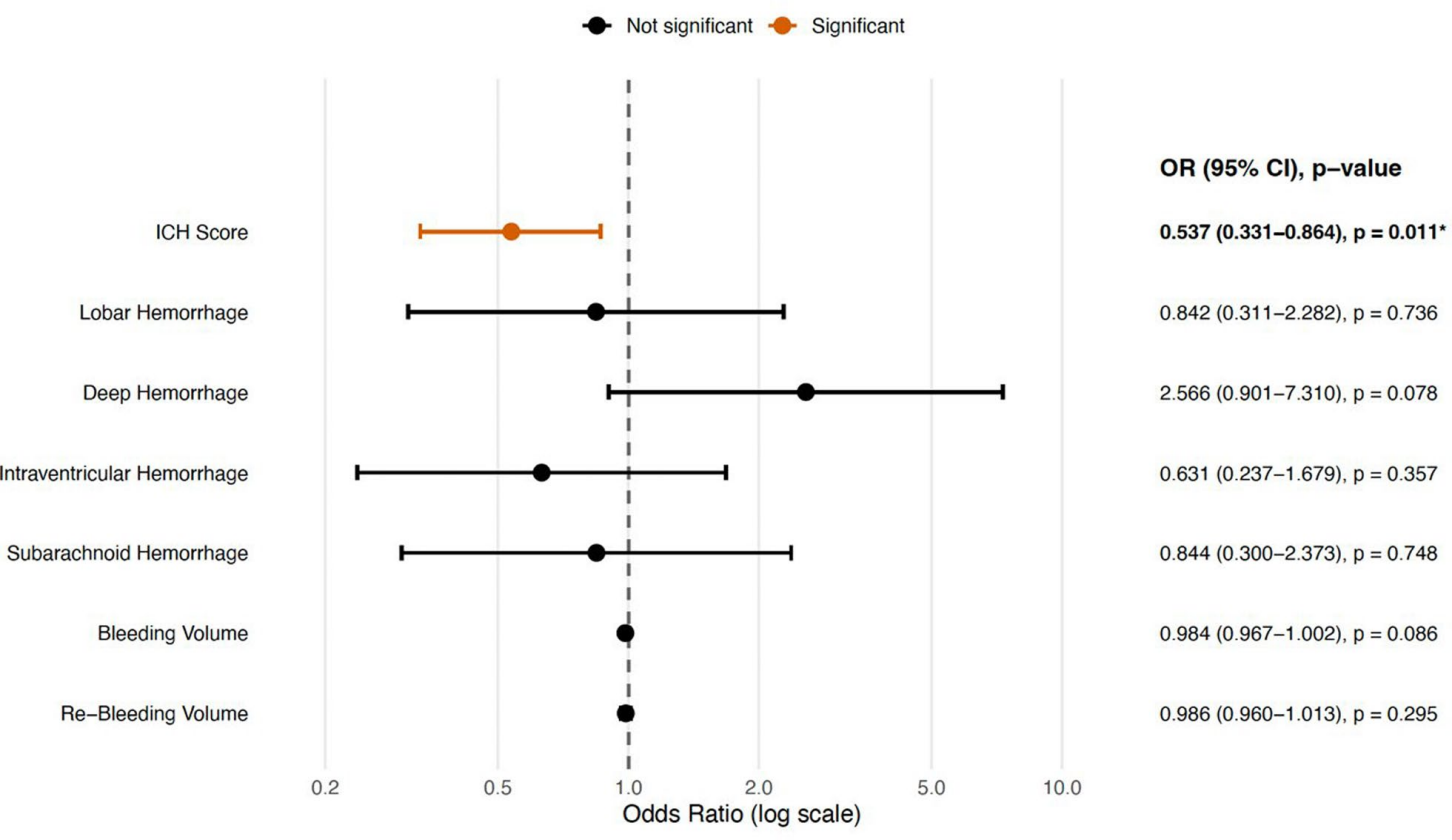
Forest plot of predictors of good outcome after FEES. Points show odds ratios and horizontal lines show 95% confidence intervals

In FEES-filtered analyses (patients who underwent FEES), higher ICH score predicted lower odds of a good FOIS-G before FEES (aOR 0.535, 95% CI 0.326–0.876; p = 0.013) and after FEES (aOR 0.645, 95% CI 0.422–0.985; p = 0.034; confidence intervals back-transformed from log-odds where available) (*refer to *Fig. 2)*.* For FEDSS (good outcome), a higher ICH score was likewise associated with lower odds (aOR 0.537, 95% CI 0.331–0.864; p = 0.011) (*refer to *Fig. 3)*.*

### Correlations between dysphagia assessment outcomes and clinical, functional, and radiologic measures

In the overall dysphagia cohort, a higher FOIS-G (> 6) after the dysphagia assessment correlated with better functional status and lower neurological severity: higher Barthel index at discharge (r = 0.54, p < 0.001), higher GCS on admission (r = 0.39, p < 0.001), and lower NIHSS both at admission (r = − 0.45, p < 0.001) and discharge (r = − 0.53, p < 0.001). A higher FOIS-G after the assessment was also associated with shorter hospital stay (r = − 0.22, p = 0.004) and fewer days of mechanical ventilation (r = − 0.37, p < 0.001). Radiologic severity showed inverse relationships: higher ICH score (r = − 0.30, p < 0.001), larger hematoma volume (r = − 0.19, p = 0.013), and higher Graeb score (r = − 0.23, p = 0.002) were each linked to a lower probability of a higher FOIS-G after the assessment. Intraventricular extension (r = − 0.23, p = 0.003) and concomitant SAH (r = − 0.20, p = 0.010) were likewise unfavourable, whereas a small positive signal was observed for occipital lobar location (r = 0.19, p = 0.015).

In FEES-filtered analyses, a higher FOIS-G after FEES closely tracked lower FEDSS (r = 0.83, p < 0.001) and also related to better function (Barthel at discharge: r = 0.33, p = 0.011) and lower neurological severity (NIHSS at discharge: r = − 0.30, p = 0.020; ICH score: r = − 0.25, p = 0.038). For FEDSS low) specifically, correlations pointed in the same direction: a lower FEDSS was associated with higher Barthel at discharge (r = 0.29, p = 0.025), smaller hematoma volume (r = − 0.25, p = 0.046; ρ = − 0.30, p = 0.015), and lower ICH score (r = − 0.31, p = 0.009). Rank-based testing also suggested an inverse association between FEDSS and rebleed volume (ρ = − 0.30, p = 0.011) and between FOIS-G after FEES and rebleed volume (ρ = − 0.25, p = 0.045) where Pearson’s tests were not significant.

In this cohort, patients who underwent FEES were clinically more severely affected than patients without FEES. The FEES group had a longer hospital stay (median 25 [IQR 14–32] vs 12 [5–20] days; *p* < 0.001), a higher NIHSS at discharge (median 8 [4–12] vs 4 [1–10]; *p* < 0.001), and a longer duration of mechanical ventilation (median 240 [IQR 120–527] vs 120 [48–408]; *p* = 0.013). They also showed higher rates of pneumonia (63.2% vs 35.8%; *p* < 0.001) and PEG placement (27.9% vs 12.1%; *p* = 0.006) (Table 3).

**Table 3:** Secondary FEES-related outcomes. Abbreviations: PEG = Percutaneous Endoscopic Gastrostomy; LoS = Length of hospital stay; NIHSS = National Institute Hospital Stroke Scale

	FEES (n = 68)	No FEES (n = 173)	p-Value
PEG	19 (27.9)	21 (12.1)	,006
Pneumonia	43 (63.2)	62 (35.8)	<,001
LoS	25 (14;32)	12 (5;20)	<,001
NIHSS at discharge	8 (4;12)	4 (1;10)	<,001
Duration of mechanical ventilation	240 (120;527)	120 (48; 408)	,013
Mechanical ventilation	31 (45.6)	71 (41.0)	,564
Tracheotomy	9 (13.2)	19 (11.0)	,657

## Discussion

This study demonstrates that dysphagia is common after intracerebral haemorrhage and closely associated with radiologic severity and lesion characteristics. *Taken together, our findings suggest that radiologic severity and lesion characteristics are associated with swallowing impairment after ICH, while FEES frequently provides additional information leading to clinically relevant adjustments in swallowing management. Thus, neuroimaging and FEES may offer complementary value for dysphagia risk stratification and clinical decision-making in this population*.

Approximately half of all ICU-treated ICH patients in our cohort were diagnosed with relevant dysphagia, which is consistent with previous reports using both clinical and instrumental diagnostics [3—5]. Importantly, FEES identified clinically meaningful discrepancies between bedside DA and actual swallowing function. In two thirds of patients undergoing FEES, swallowing management was modified, in most cases allowing safer and more liberal oral intake. This finding explains the observed increase in FOIS-G scores after FEES and highlights the clinically relevant divergence between clinical and instrumental dysphagia diagnostics [1, 16, 17]*.* Importantly, these differences should be interpreted as reflecting baseline severity and a more complicated clinical course among patients selected for FEES, not as outcomes caused by FEES itself.

Radiologic burden emerged as a central determinant of DS. Higher ICH scores, deep hemorrhage location, intraventricular hemorrhage (IVH), and subarachnoid hemorrhage (SAH) were independently associated with worse swallowing outcomes as measured by both FEDSS and FOIS-G. These findings support the concept that dysphagia after ICH is primarily driven by disruption of strategic swallowing-related networks rather than hematoma size alone [3, 6—8]. Lesions involving subcortical structures, opercular regions, or thalamocortical pathways may impair sensorimotor integration essential for safe swallowing, even when hemorrhage volume is modest [18].

Our results align with and extend previous neuroimaging studies demonstrating that lesion location plays a decisive role in post-ICH dysphagia. Strategic involvement of the insular cortex, basal ganglia, internal capsule, corona radiata, and thalamus has been associated with swallowing impairment, highlighting dysphagia as a network phenomenon rather than a simple marker of global disease severity [3, 6—8]. Clinically, this underscores that even small hemorrhages in critical locations may warrant early dysphagia evaluation [3, 6, 7, 8]*.*

Beyond radiologic predictors, clinical severity indices such as NIHSS, GCS, and overall functional outcome were closely correlated with DS, confirming prior observations from studies relying on non-instrumental diagnostics [3, 4, 5]. IVH and SAH likely contribute to dysphagia through secondary mechanisms including impaired consciousness, hydrocephalus, prolonged mechanical ventilation, and the need for invasive interventions [4, 5, 6].

From a clinical perspective, our findings have implications that extend beyond specialized neuro-ICUs. Radiologic markers such as ICH score, hemorrhage location, and intraventricular extension are readily available in all acute care settings. *Previous studies in spontaneous intracerebral haemorrhage have established the prognostic relevance of simple baseline clinical and CT-based variables, whereas our findings suggest that these routinely available markers may also help identify patients at risk of clinically significant swallowing impairment.* Imaging-informed dysphagia risk stratification may therefore guide early referral for instrumental swallowing evaluation or justify precautionary feeding strategies in hospitals without on-site swallowing specialists [26].

Several limitations merit consideration. FEES was performed in only a subset of dysphagic patients, reflecting real-world feasibility constraints and introducing selection bias toward clinically stable individuals. Due to the retrospective design and limited sample size of the FEES subgroup, formal comparisons of outcomes such as pneumonia or long-term feeding-tube dependency between patients with and without FEES were not statistically robust. Moreover, the absence of long-term follow-up data precludes conclusions regarding causal effects of FEES-guided management on functional recovery.

Despite these limitations, this study represents one of the largest FEES-based analyses of dysphagia after intracerebral haemorrhage and provides novel insight into the interaction between radiologic injury patterns and swallowing function.

## Data Availability

The datasets generated and/or analysed during the current study are not publicly available due to agreements required for ethics, data protection and privacy, but are available from the corresponding author on reasonable request.
